# Supplementary material for: TGF-β Isoforms Affect the Planar and Subepithelial Fibrogenesis of Human Conjunctival Fibroblasts in Different Manners
Source: Biomedicines. 2023 Jul 15;11(7):2005. doi: 10.3390/biomedicines11072005 (PMC10377695; doi:10.3390/biomedicines11072005)
Supplement: Supplementary file 1 [file biomedicines-11-02005-s001.zip › biomedicines-2442727-supplementary.pdf]

Supplemental Table S1. Sequences of primers of qPCR

		Sequence	Exon Location	RefSeq Number
human RPLP0[*1]	Probe	5'-/56-FAM/CCCTGTCTT/ZEN/CCCTGGGCATCAC/3IABkFQ/-3'	2-3	NM_001002
	Primer2	5'-TCGTCTTTAAACCCTGCGTG-3'		
	Primer1	5'-TGTCTGCTCCCACAATGAAAC-3'		
human COL1A1[*1]	Probe	5'-/56-FAM/TCGAGGGCC/ZEN/AAGACGAAGACATC/3IABkFQ/-3'	1-2	NM_000088
	Primer2	5'-GACATGTTCAGCTTTGTGGAC-3'		
	Primer1	5'-TTCTGTACGCAGGTGATTGG-3'		
human COL4A1[*1]	Probe	5′-/56-FAM/TCATACAGA/ZEN/CTTGGCAGCGGCT/3IABkFQ/-3′	51-52	NM_001845
	Primer2	5′-AGAGAGGAGCGAGATGTTCA-3′		
	Primer1	5′-TGAGTCAGGCTTCATTATGTTCT-3′		
human COL6A1[*1]	Probe	5′-/56-FAM/CAGGTTTCG/ZEN/GTCACAGCGGTAGT/3IABkFQ/-3′	2-3	NM_001848
	Primer2	5'-CCTCGTGGACAAAGTCAAGT-3'		
	Primer1	5'-GTGAGGCCTTGGATGATCTC-3'		
human FN1[*1]	Probe	5′-/56-FAM/TACAGCTTA/ZEN/TTCTCCCTCGCCCAG/3IABkFQ/-3′	3-4	NM_212482
	Primer2	5'-CGTCCTAAAGACTCCATGATCTG-3'		
	Primer1	5'-ACCAATCTTGTAGGACTGACC-3'		
human αSMA[*1]	Probe	5'-/56-FAM/AGACCCTGT/ZEN/TCCAGCCATCCTTC/3IABkFQ/-3'	8-9	NM_001613
	Primer2	5'-AGAGTTACGAGTTGCCTGATG-3'		
	Primer1	5'-CTGTTGTAGGTGGTTTCATGGA-3'		
